# E-FABP: regulator of immune function

**DOI:** 10.18632/oncoscience.54

**Published:** 2014-06-15

**Authors:** Yuwen Zhang, Bing Li

**Affiliations:** The Hormel Institute, University of Minnesota, Austin, MN

Fatty acids are essential for many aspects of biological activities. They function not only as an energy source but also as signaling molecules, regulating immune responses and other vital cellular functions. However, how to control fatty acid trafficking while sustaining lipid homeostasis inside cells remains largely unknown. As the main cytoplasmic lipid chaperones, fatty acid binding proteins (FABPs) are known to bind a variety of fatty acids and endogenous hydrophobic metabolites, facilitating lipid transportation and coordinating their responses [1]. Thus, FABPs are believed to play a central role in mediating metabolic balance and lipid-mediated responses through the regulation of diverse lipid signals.

The FABP family consists of at least nine members of highly homologous proteins, each of which has been named according to the tissue where they were first cloned, such as adipose FABP (A-FABP) and intestinal FABP (I-FABP) [2]. While most FABP members display tightly-regulated patterns of tissue distribution, we noticed that epidermal FABP (E-FABP) is widely expressed in skin epidermal cells, vessel endothelial cells, and organ epithelial cells. Moreover, E-FABP is the predominant member of the FABP family which exists in various types of immune cells [3]. This ubiquitous expression pattern suggests that E-FABP is critical in maintaining cellular basic energy metabolism, thereby contributing to epithelial integrity and immune cell functions.

In our research focusing on the role of E-FABP in tumor development, we found that mice deficient for E-FABP exhibited increased tumor growth and metastasis in different tumor models as compared to their wild-type counterparts [4]. To dissect the mechanisms of E-FABP in suppression of mammary tumor growth we have identified that tumor associated macrophages (TAMs), in particular a specific subset of TAMs which exhibit the CD11b^+^ F4/80^+^ CD11c^+^ MHCII^+^ phenotype, highly express E-FABP and play a pivotal role in E-FABP-mediated protection against mammary tumor growth and metastasis. Furthermore, microarray analysis of genes expressed differentially in wild-type and E-FABP^−/−^ macrophages demonstrated that E-FABP expression in TAMs strengthens interferon β (IFNβ) production and signaling. The E-FABP-regulated IFNβ responses can further recruit the infiltration of natural killer (NK) cells into the tumor microenvironment to enhance tumor killing activity. Thus, E-FABP is critical in regulating host immune responses to tumor challenges, and host expression of E-FABP may represent a new protective factor towards cancer prevention through enhancing anti-tumor activity of TAMs.

However, everything has two sides. While host expression of E-FABP is able to inhibit tumor growth and metastasis, E-FABP overexpression has been shown to promote inflammatory autoimmune diseases. In a mouse model of human multiple sclerosis, upregulation of E-FABP has been demonstrated to promote the development of experimental autoimmune encephalomyelitis (EAE). On the one hand, E-FABP expression in macrophages and dendritic cells facilitates the production of inflammatory cytokines including IL-6, TNFα, IL-1β, etc [5]. On the other hand, T cell expression of E-FABP can increase Th17-cell differentiation, while decreasing the development of regulatory T cells (Tregs) [6]. Thus, E-FABP is critical in shaping both innate and adaptive immune responses for EAE development. In line with those observations, we further demonstrated that consumption of a high-fat diet upregulates E-FABP expression in macrophages and keratinocytes in skin tissues, which significantly instigates inflammatory skin lesions in a mouse model of obesity. In contrast, mice deficient for E-FABP are completely resistant to high-fat diet-induced skin inflammation (Zhang, unpublished data). Altogether, our data suggest E-FABP plays a dual role regarding the regulation of immunological functions. While E-FABP expression is indispensable for effective tumor immune surveillance, aberrant E-FABP activity may overactivate immune cells leading to inflammatory autoimmune diseases.

In exploring the molecular mechanisms of how E-FABP expression in immune cells regulates their differentiation and functions, we observed several interesting phenomena. (1) Macrophages deficient for E-FABP exhibit reduced activation of STAT1 and STAT2 in response to IFNβ stimulation. (2) E-FABP can modulate gene expression through impacting the activity of nuclear transcriptional factors. For example, E-FABP expression in T cells can bind and restrict PPARγ ligands in cytoplasm, thus suppressing PPARγ activation. (3) Most importantly, E-FABP-mediated lipid transportation can facilitate immune cells to establish essential lipid platforms (e.g. lipid droplets), which bind specific signaling proteins for the regulation of cytosolic immune responses [7]. All the evidence supports the idea that E-FABP represents a new regulator by coupling metabolic and inflammatory pathways in immune cells.

In summary, integration of documented research and our studies indicate that E-FABP plays a unique role in regulating immune responses in different diseases. Selective enhancing E-FABP activity in macrophages may represent a novel strategy for tumor prevention and treatment. Meanwhile, it should not be omitted the potential influence of E-FABP overactivity-mediated inflammatory autoimmune diseases.
